# *Dactylosporangium luridum* sp. nov., *Dactylosporangium luteum* sp. nov. and *Dactylosporangium salmoneum* sp. nov., nom. rev., isolated from soil

**DOI:** 10.1099/ijs.0.056390-0

**Published:** 2013-09

**Authors:** Byung-Yong Kim, James E. M. Stach, Hang-Yeon Weon, Soon-Wo Kwon, Michael Good

This paper was published with an incorrect doi. The correct doi is: 10.1099/ijs.0.016451-0.

